# Supplementary material for: Molecular Detection of Tetracycline-Resistant Genes in Multi-Drug-Resistant Escherichia coli Isolated from Broiler Meat in Bangladesh
Source: Antibiotics (Basel). 2023 Feb 20;12(2):418. doi: 10.3390/antibiotics12020418 (PMC9952414; doi:10.3390/antibiotics12020418)
Supplement: Supplementary file 1 [file antibiotics-12-00418-s001.zip › Table S3.pdf]

Supplementary Table S3:

Zone of Inhibition of the antimicrobial agents to classify as susceptible and resistance to *E. coli* (1)

Antimicrobial Agents	Zone of Inhibition (mm)		
	Susceptible	Intermediate	Resistance
Cephalexin (CL, 30 µg)	≥15	-	≤14
Ampicillin (AMP, 10 µg)	≥17	14 -16	≤13
Tetracycline (TE, 30 µg)	≥15	-	≤11
Doxycycline (DO,30 µg)	≥14	-	≤10
Gentamicin (CN,10 µg)	≥17	-	≤14
Ciprofloxacin (CIP,5 µg)	≥31	21 - 30	≤20
Sulphamethoxazole trimethoprim (SXT, 25 µg)	≥16	11 -15	≤10

Reference:

1. CLSI. Performance Standards for Antimicrobial Susceptibility Testing 2020 [Available from: https://www.nih.org.pk/wp-content/uploads/2021/02/CLSI-2020.pdf.
